# Time-resolved early-to-late Gadolinium enhancement MRI using single breath-hold 3D spiral imaging

**DOI:** 10.1186/1532-429X-14-S1-P250

**Published:** 2012-02-01

**Authors:** Taehoon Shin, Dwight G Nishimura, Michael V McConnell, Bob S Hu

**Affiliations:** 1 Electrical Engineering, Stanford University, Stanford, CA, USA; 2 Cardiovascular Medicine, Stanford University, Stanford, CA, USA; 3 Palo Alto Medical Foundation, Palo Alto, CA, USA; 4 Heart Vista Inc., Palo Alto, CA,USA

## Background

The assessment of myocardial infarction (MI) heterogeneity using late Gadolinium enhancement (LGE) MRI has been limited to static analysis at single late post-injection time. We developed a rapid single breath-hold LGE MRI method using a novel parallel imaging accelerated 3D spiral acquisition. We propose to use this technique to capture temporal variation in scar enhancement at early to late post-injection times, termed early-to-late Gadolinium enhancement (ELGE) MRI.

## Methods

We developed an inversion recovery 3D spiral imaging sequence with a novel iterative self-consistent parallel imaging reconstruction (SPIRiT) method, which allows entire LV coverage within only 12 heart beats. The scan parameters were: acceleration factor = 2, TI=200-300ms, spatial resolution = 1.7×1.7×7 mm3, 14 partition slices, 190 ms data acquisition window at mid-diastole, a GE 1.5 T scanner, and an 8-channel cardiac coil. Starting at 1 or 2 minutes after contrast administration, the 3D ELGE imaging was performed every minute until 10 minutes post injection. Afterwards, the standard 2D multi-slice LGE MRI was performed. The time series of 3D ELGE data were registered using a mutual information-based motion correction to compensate for different breath-hold positions.

## Results

**Figure 1:**
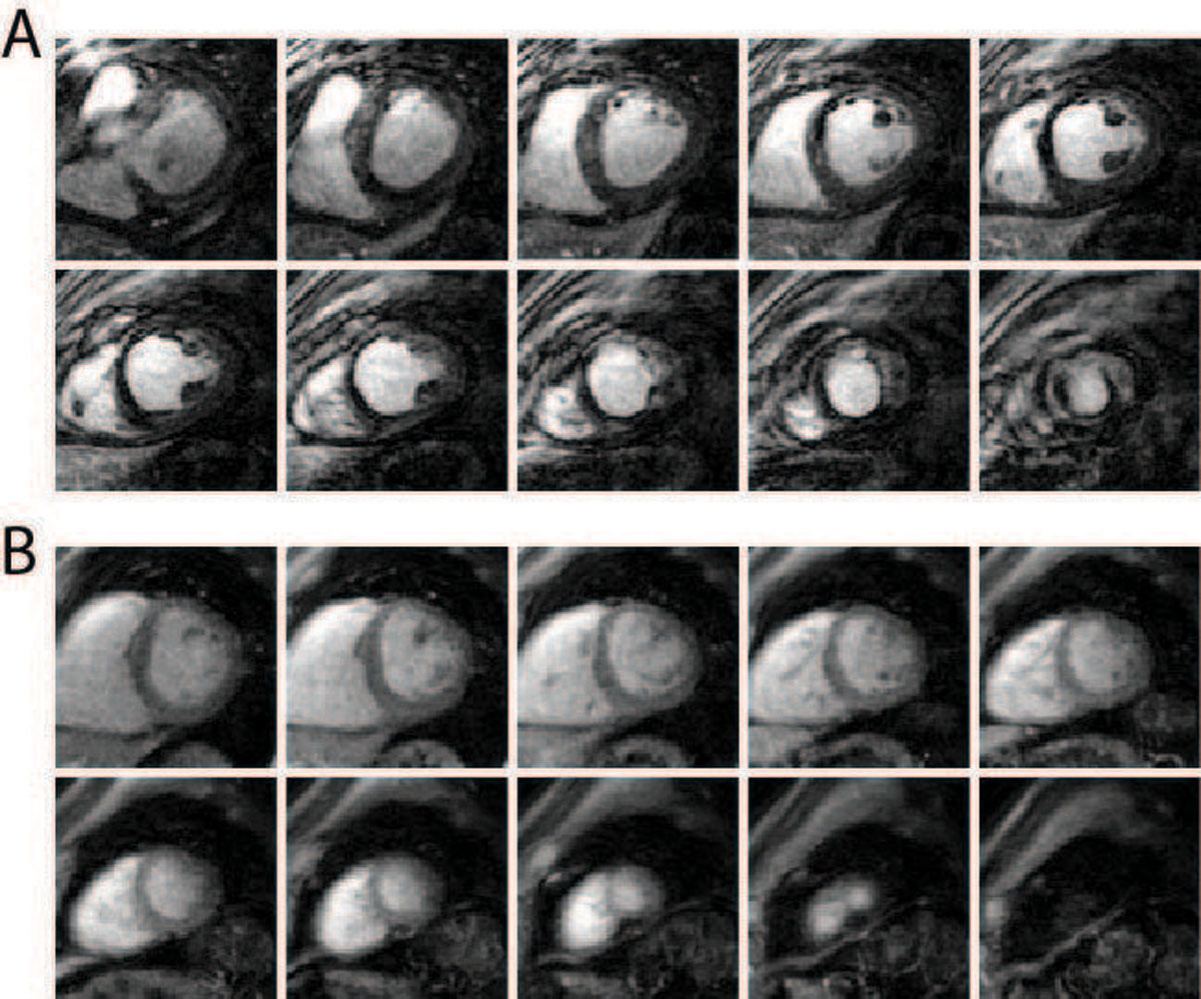
Representative 3D ELGE images taken at 2 minutes after contrast administration in patients with scar (A) and without scar (B). A: The region of scar on anteroseptal wall appears darker than the remote region due to lower perfusion. B: Contrast enhancement is homogeneous over entire myocardium.

**Figure 2:**
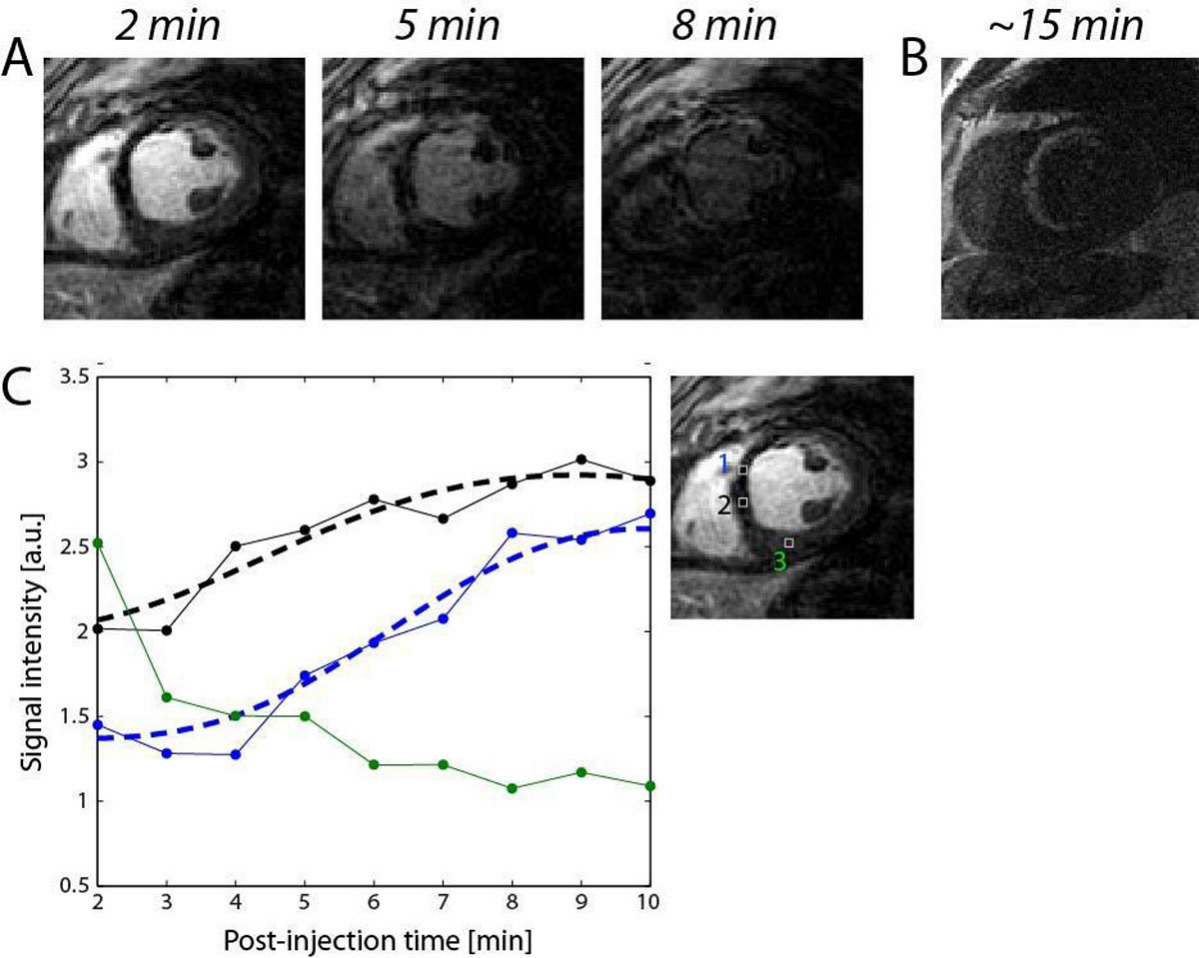
A: Mid-short-axis slice of 3D ELGE images acquired at post-injection times of 2 min, 5 min, and 8 min from a patient with MI. B: 2D image from the commercial LGE sequence at the same slice location. C: Signal intensities in the regions of MI increases over time at different enhancement rates (ROIs 1 and 2) whereas the intensity in the ROI of normal myocardium decreases over time (ROI 3).

Figure 1 shows representative 3D ELGE images taken at 2 min post-injection time from two subjects (A: with MI, B: without MI). Figure 1A shows hypo-enhancement in the scar region due to lower perfusion of contrast agent whereas Fig. 1B exhibits homogeneous intensities over entire myocardium. Signal intensities in the scar region gradually increase over time, but at different levels and rates depending on spatial position and post-injection time (Fig. 2A). This temporal variation in the scar enhancement is lost in the conventional 2D LGE image that was acquired at ~15 min post injection time (Fig. 2B). The scar heterogeneity is demonstrated also by time intensity curves of user-defined ROIs as shown in Fig. 2C (ROIs 1 and 2 from the scar region, ROI 3 from the remote region). This highlights that the intensity of the scar at ROI 1 is lower at early enhancement and starts to increase later than the scar at ROI 2.

## Conclusions

Time-resolved characterization of myocardial scar tissue is feasible using 3D spiral ELGE MRI. Visual assessment and ROI-based time curve analysis demonstrated that scar enhancement is heterogeneous both spatially and temporally. Clinical validation of the proposed ELGE MRI in a large cohort of MI patients remains to be investigated.

## Funding

AHA postdoctoral fellowship (09POST2150025).

NIH SBIR grant (5R44HL084769).

